# In-vitro characterization and evaluation of mesoporous titanium dioxide composite hydroxyapatite and its effectiveness in occluding dentine tubules

**DOI:** 10.1186/s12903-021-01989-z

**Published:** 2022-02-23

**Authors:** Lu Yin, Xuehong Xu, Chienyu Chu, Pingting Lin, Honglan Huang, Bizhu Luo, Changwei Yang

**Affiliations:** 1 Xiamen Key Laboratory of Stomatological Disease Diagnosis and Treatment, Stomatological Hospital of Xiamen Medical College, No. 1309, Lvling Road, Huli District, Xiamen, 361008 Fujian China; 2 Engineering Research Center of Fujian University for Stomatological Biomaterials, Xiamen, 361008 Fujian China

**Keywords:** Titanium dioxide, Hydroxyapatite, Dentin hypersensitivity, Occlusion

## Abstract

**Background:**

To synthesize mesoporous titanium dioxide composite hydroxyapatite (TiO_2_–HAP) and to evaluate its effectiveness in sealing of occluding dentine tubules.

**Methods:**

TiO_2_–HAP was synthesized by chemical precipitation method and characterized using infrared absorption spectrometer, X-ray diffraction, scanning electron microscope, and specific surface area detector. Forty completely extracted molars were prepared and randomly assigned into Control group, Gluma group, HAP group and TiO_2_–HAP group according to different treatments. The characteristics of HAP and TiO_2_–HAP and the sealing effectiveness of dentine tubules in these four groups, including infrared spectrum, surface contact angle, pore size distribution, and re-mineralized enamel surface profiles, were analyzed by suitable characterized techniques. The cytotoxicity of the synthesized TiO_2_–HAP was tested and compared using 3-(4,5)-dimethylthiahiazo(-z-y1)-3,5-di-phenytetrazoliumromide (MTT) colorimetry.

**Results:**

Our results showed TiO_2_–HAP group had significantly lower contact angle, higher specific surface area, and wider range of pore size distribution than other groups. The majority of dentinal tubules in the TiO_2_–HAP group were blocked by white matter in a uniformed manner, and the crystals arranged in order grew along the axial direction. In addition, no significant difference in optical density (OD) value was found between control group and TiO_2_–HAP group (*P* > 0.05), and cell growth was good in TiO_2_–HAP group, indicating no cytotoxicity of TiO_2_–HAP.

**Conclusions:**

The MTT assay identified that TiO_2_–HAP had little effect on the L929 cell line. We showed TiO_2_–HAP might be used as a remineralization agent in enamel caries-like lesions.

## Background

Dentine hypersensitivity (DH) is a significant public oral health concern with a severe consequence to individual social life, psychological status, and/or discomfort from pain, affecting over 43% of adult’s population globally [1]. The underlying mechanism for DH actions is still awaiting investigation, and the widely accepted hydrodynamic theory suggested that fluid movement along the dentin tubules resulted from external stimuli activates the terminals of the pulp and cause pain [2]. In this scenario, occlusion of dentin tubules by natural or artificial materials might be an effective approach in the treatment of DH since it could render the movement of intratubular flows through mechanical manner [3].

A large variety of different occlusion materials, such as hydroxyapatite (HAP), potassium oxalates, sodium fluoride, calcium glycerophosphate, and abrasive agents, have been investigated for the treatment of DH in oral care industry [4]. Among which, nanohydroxyapatite, as one of the most biocompatible and bioactive materials, has extensively been applied in dentistry due to its favorable properties such as similarities to the mineral phase of the human hard tissues, biocompatibility, and low solubility in humid environments [5]. Indeed, it has been suggested in previous study that nanohydroxyapatite could be applied to repair dental enamel and treat DH [6].

In addition, as an n-type semiconductor, mesoporous titanium dioxide (TiO_2_) has extensive applications in photocatalysis and conversion of solar energy because of its high refractive index, wide band gap, chemical stability, and low cost [7]. With the structure of hollow spheres, TiO_2_ have attracted extensive attention in the fields of catalysis, microreactors, adsorption, and drug delivery [7]. For instance, the large fraction of void space in hollow structures has been successfully used to encapsulate and control release of sensitive materials such as drugs, cosmetics, and DNA [8]. Therefore, we proposed that incorporating HAP into nanoscale mesoporous TiO_2_ composite (mesoporous TiO_2_ composite HAP, TiO_2_–HAP) using chemical precipitation method might produce a fabricate biomaterial with suitable surface to volume ratio, antibacterial action, physical, mechanical, biological characteristics, and distinctive particle size, which renders it effective vehicles for dental applications. The characteristics of TiO_2_–HAP and HAP, and their occluding effectiveness of dentine tubules were analyzed by suitable characterized techniques.

## Methods

### Materials

Calcium nitrate (Ca (NO_3_)_2_), potassium nitrate (KNO_3_), and ammonia (NH_3_·H_2_O) were purchased from Guoyao Chemical Reagent Co., Ltd. (Shanghai, China). Diammonium hydrogen phosphate ((NH_4_)_2_HPO_4_) was purchased from Xilong Chemical Co., Ltd. (Shantou, China). X-ray diffractometer (D/max RB) was obtained from Japanese Neo Confucianism motor. Infrared absorption spectrometer (SPECTRO LAB M11) was obtained from SPEK analytical instruments Co., Ltd. Scanning electron microscope (SEM)/energy dispersive spectrometer (JSM-6510) and transmission electron microscope (JEM-1400plus) were bought from Japan Electronics Co., Ltd. Specific surface area detector (F-Sorb 2400) was obtained from Beijing Kingaipu Technology Co., Ltd. HAP composites containing mesoporous TiO_2_ and HAP materials without TiO_2_ were both self-made.

### Preparation of TiO_2_–HAP

Mesoporous TiO_2_–HAP was synthesized by chemical precipitation method as previously described [3]. In general, 5 ml tetrabutyl titanate (TBOT), 2 ml acetylacetone and 3.14 mg stearic acid were mixed and stirred for 30 min to obtain the TBOT complex, which was then slowly dispersed into 40 ml deionized water under the condition of rapid stirring, and a milky yellow dispersion system was formed by continuously stirring for 30 min. The dispersion system was then transferred to three polytetrafluoroethylene reactors with a volume of 25 ml, and then reacted in water for 10 h at 150 °C. The products were centrifugally washed with distilled water and absolute ethanol, respectively. Finally, the product was dried in an oven at 80 °C for 6 h to obtain TiO_2_–HAP.

### Preparation of molar teeth samples

Forty molars were collected and the residual soft tissue around the teeth was removed. The teeth were cleaned by ultrasonic cleaning and stored in 0.1% thymol solution at 4 °C. Perpendicular to the long axis of the tooth, the root was excised about 2 mm away from the enamel cementum boundary root. The enamel of occlusal surface was completely removed and dentin was exposed. After wetting the dentin surface, the dentin surface was polished with silicon carbide water sandpaper to prepare a standard bonding surface of 3 mm × 3 mm × 2 mm, and ultrasonic cleaning was performed for 10 min. The surface of teeth was dried and divided into control group, Gluma group, HAP group and TiO_2_–HAP group (10 sample/group) (Table 1). The sealing effect was observed under scanning electron microscope (SEM) when the samples were coated and placed for 1 week.

**Table 1:** Sample grouping and preservation conditions

Groups	Treatment method	Preservation conditions
Control group	The sample surface was not treated	After being embedded in wax, it was stored at 37 °C for 7 days
Gluma group	Dry the sample, dip it in Gluma desensitizer, and smear the sample surface repeatedly for 10 min	
HAP group	Dry the sample, dip it in HAP desensitizer, and smear the sample surface repeatedly for 10 min	
TiO_2_–HAP group	Dry the sample, dip it in TiO_2_–HAP desensitizer, and smear the sample surface repeatedly for 10 min	

### Characterization of TiO_2_–HAP and other materials

#### Infrared spectroscopy analysis

To analyze the chemical components of samples, the infrared spectra were measured using a Perkin Elmer Universal ATR spectrometer to identify the functional group constituents of TiO_2_–HAP and HAP.

#### X-ray diffraction analysis

The X-ray diffraction (XRD) analysis was performed to observe the possible changes in phase between the different samples. The XRD patterns were recorded using a diffractometer (PANalytical-Empyrean instrument. Co.) and analyzed between 0 and 90 °C (2 theta). The voltage, current and pass time used were 40 kV, 40 mA and 1 s, respectively.

#### High-resolution transmission electron microscopic analysis

The morphology of the prepared samples was observed under a high-resolution transmission electron microscope (HRTEM; Philips CM 120 model) according to the published article [4].

#### F-Sorb 2400 surface area analyzer

According to the Brunauer Emmett Teller equation theory and the Barrett Joyner Halenda method, the specific surface area and total pore volume of HAP and TiO_2_–HAP were measured by F-Sorb 2400 surface area analyzer with the exhaust temperature of 120 °C and the degassing time of 45 min [9]. Because of the very small diameter of nitrogen molecule, nitrogen adsorption method was used. It was measured by the inherent droplet method. A micropipette was used to take 0.5 µl of the liquid onto the surface of the four samples. After 5 s, each sample can be photographed and recorded at three optional points. Then change the specimen and test liquid in turn. After developing the film, enlarge the photo by 3.5 times and measure the contact angle of each time with a goniometer. The average contact angle of three points of each specimen was taken as the contact angle of this specimen. According to the contact angle, the surface energy was calculated by Mathematica 4.1 software package.

#### MTT colorimetric method

The specimens were cleaned with absolute ethanol for 20 min, washed with distilled water, sterilized at 121 °C and 33 MPa, and dried in an oven. Then the specimen was placed in a glass bottle and 20 ml normal saline was added at 37 °C for 72 h. The MTT colorimetric method was carried out according to the Chinese national standard GB/T 16886.5-1997 biological evaluation of medical devices cytotoxicity test in vitro method. The OD mean value (n = 6) of each group was calculated. Cell proliferation percentage (RGR%) = (OD mean value of each concentration group/OD mean value of negative control group) × 100%. According to Table 2, the RGR% of each concentration group of the extract was converted into 0–5 grade cytotoxicity.

**Table 2:** Corresponding relationship between cell proliferation percentage and cytotoxicity grade

RGR%	Cytotoxicity grade
≥ 100	Grade 0
75–99	Grade 1
50–74	Grade 2
25–49	Grade 3
0–24	Grade 4
0	Grade 5

### Statistical analysis

Statistical analysis was made by software Statistical Product and Service Solutions (SPSS) version 17.0 (International Business Machines, corp., Armonk, NY, USA). Significant differences between groups were assessed by One-way analysis of variance (ANOVA). This was followed by a multi-comparison test with Bonferroni correction (α = 0.05). All data were expressed as means ± standard deviation (SD). Differences were considered statistically significant when *P* < 0.05.

## Results

### Sample characterization and dentin surface energy analysis

**Fig. 1:**
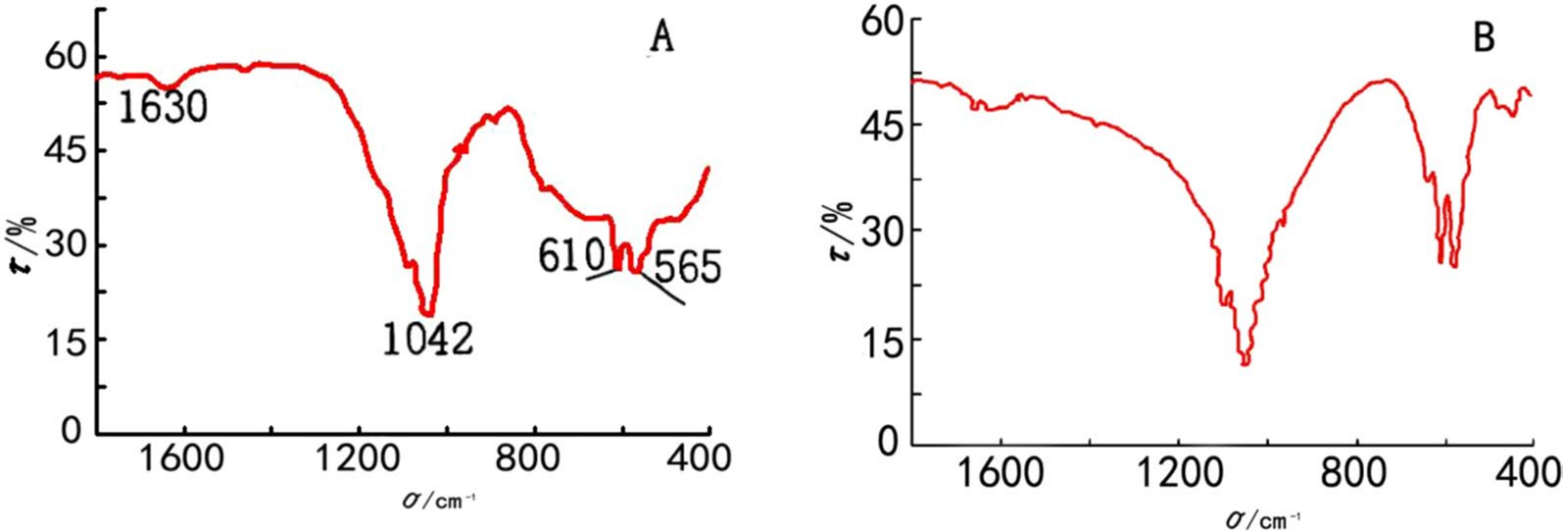
Infrared spectrum. **a** TiO_2_–HAP; **b** hydroxyapatite

**Fig. 2:**
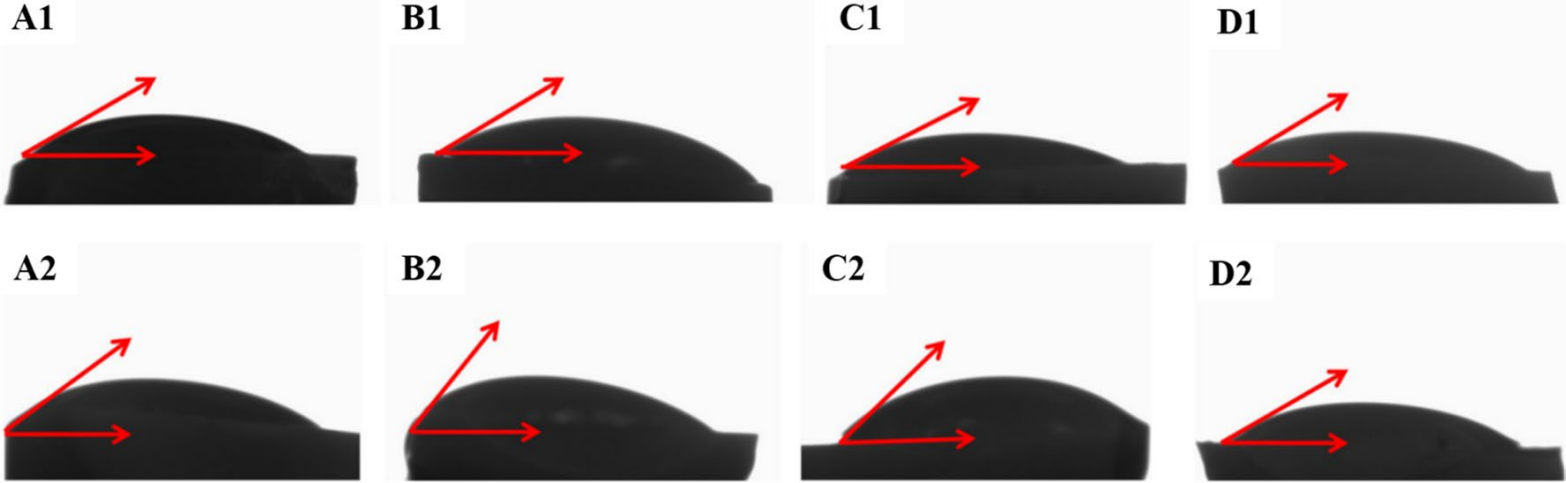
The values of contact angle. **a** Control group; **b** Gluma group; **c** HAP group and **d** TiO_2_–HAP group

As shown in Fig. 1, the infrared absorption at 1042, 605 and 560 cm^−1^ of the TiO_2_–HAP was consistent with the standard spectrum of HAP. The absorption peaks at 605 cm^−1^ and 560 cm^−1^ were obviously superimposed on the envelope peaks at 500–700 cm^−1^, which indicated that the sample contains both HAP and TiO_2_. Moreover, as shown in Table 3 and Fig. 2, the contact angles in Gluma, HAP and TiO_2_–HAP groups were significantly decreased than that in the control group in Double distilled water and Diiodiomethane (*P* < 0.05).

**Table 3:** Surface contact angle and surface free energy values

Groups	Contact angle (θ^o^)		Surface free energy (nJ/cm^2^)		
	Double distilled water	Diiodiomethane	γ_s_	γ_s_^d^	γ_s_^p^
Control group	68.78 ± 12.15	55.14 ± 10.34	44.19 ± 1.36	30.44 ± 0.65	13.75 ± 0.34
Gluma group	50.78 ± 9.14*	29.75 ± 7.16*	48.24 ± 3.56	20.40 ± 0.54	23.84 ± 0.68
HAP group	40.86 ± 18.16*	32.91 ± 8.43*	59.70 ± 3.27	47.53 ± 0.66	12.17 ± 0.54
TiO_2_–HAP group	32.78 ± 7.14*	38.75 ± 5.16*	46.24 ± 3.36	25.60 ± 0.87	20.64 ± 0.73

^*^P < 0.05

### Specific surface area of TiO_2_–HAP

**Fig. 3:**
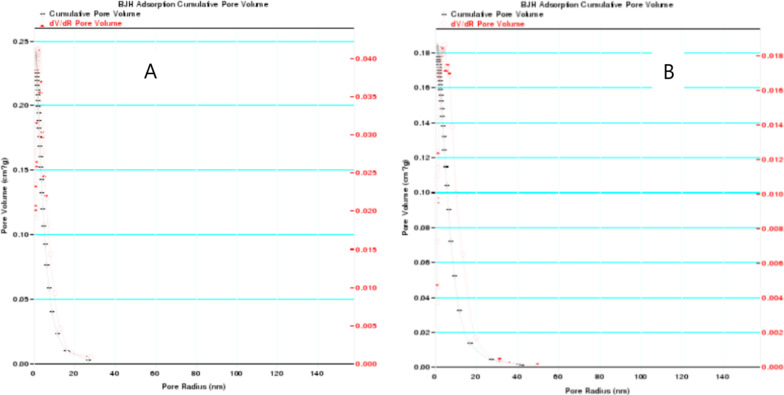
Pore size distribution curve of specimen. **a** HAP group; **b** TiO_2_–HAP group

The specific surface area in TiO_2_–HAP group was 124.65 ± 23.24 m^2^/g, and that in HAP group was 74.01 ± 11.68 m^2^/g. The total pore volume in TiO_2_–HAP group was 0.23 cm^3^/g, and that in HAP group was 0.17 cm^3^/g. Moreover, the volume pore size distribution of HAP was in the range of 0–30 nm, and that of TiO_2_–HAP was 0–50 nm, indicating wider range of pore size distribution after forming composite (Fig. 3).

### Analysis of enamel surface morphology

**Fig. 4:**
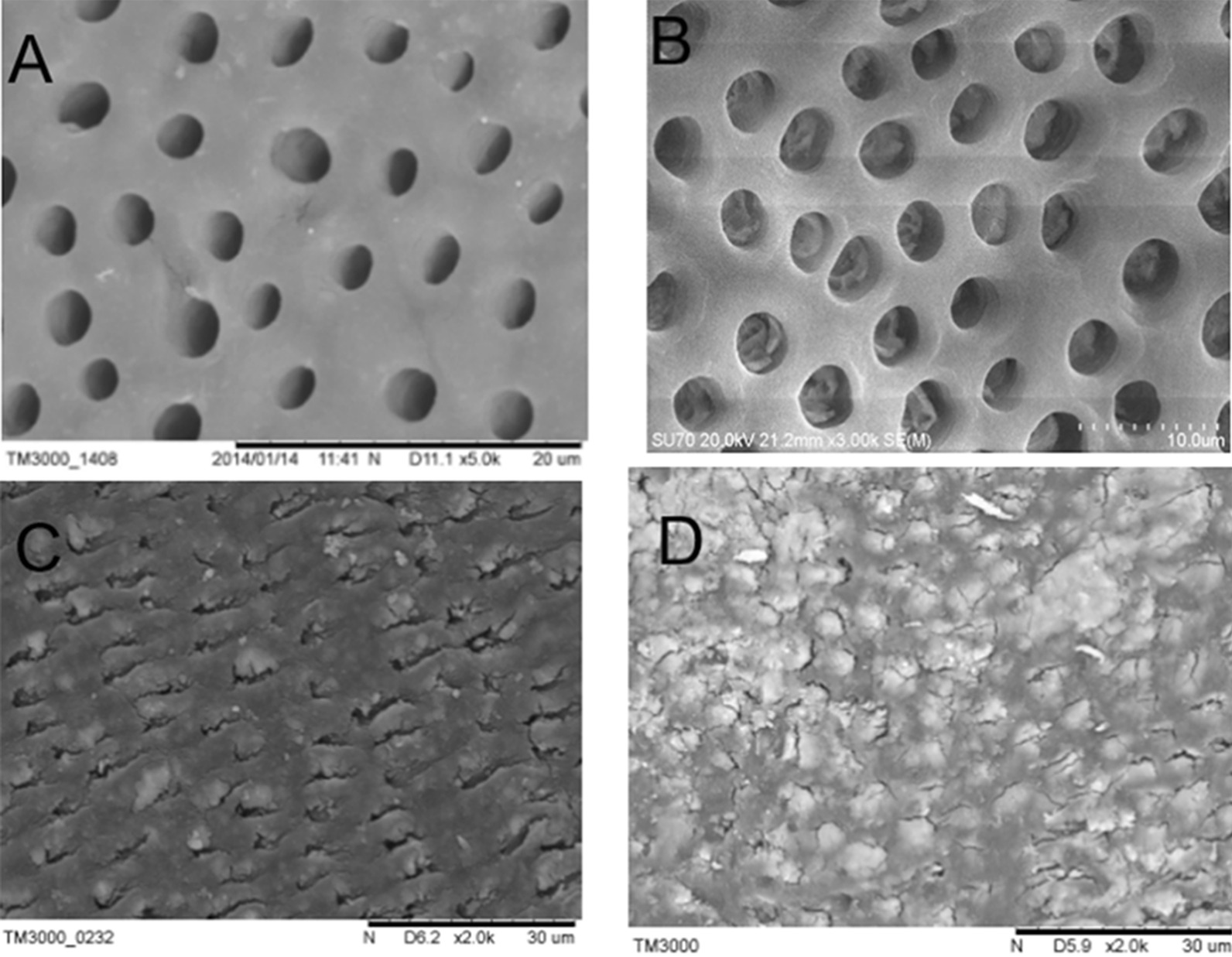
Enamel surfaces of the four groups after remineralization. **a** Control group; **b** Gluma group; **c** HAP group and **d** TiO_2_–HAP group

As shown in Fig. 4, SEM imaging showed that the dentin tubules were clearly visible, and the opening of the tubules was oval with a diameter of 1–5 µm, and the mineralization degree of periodontal dentin was higher than that of inter tubal dentin in the control group. The dentin was covered with a layer of material, and most of the canaliculi were blocked, and the diameter of the tubule orifice was significantly smaller with smooth surface in Gluma group than that in the control group. Most of the dentinal tubules were sealed by a layer of white matter, and there was a gap between the white matter and the dentin tubules, and some of the dentin tubules were not well sealed in the HAP group. Almost all the dentinal tubules were blocked by white matter, and the distribution of white matter was uniform in the TiO_2_–HAP group.

### Analysis of enamel profile

**Fig. 5:**
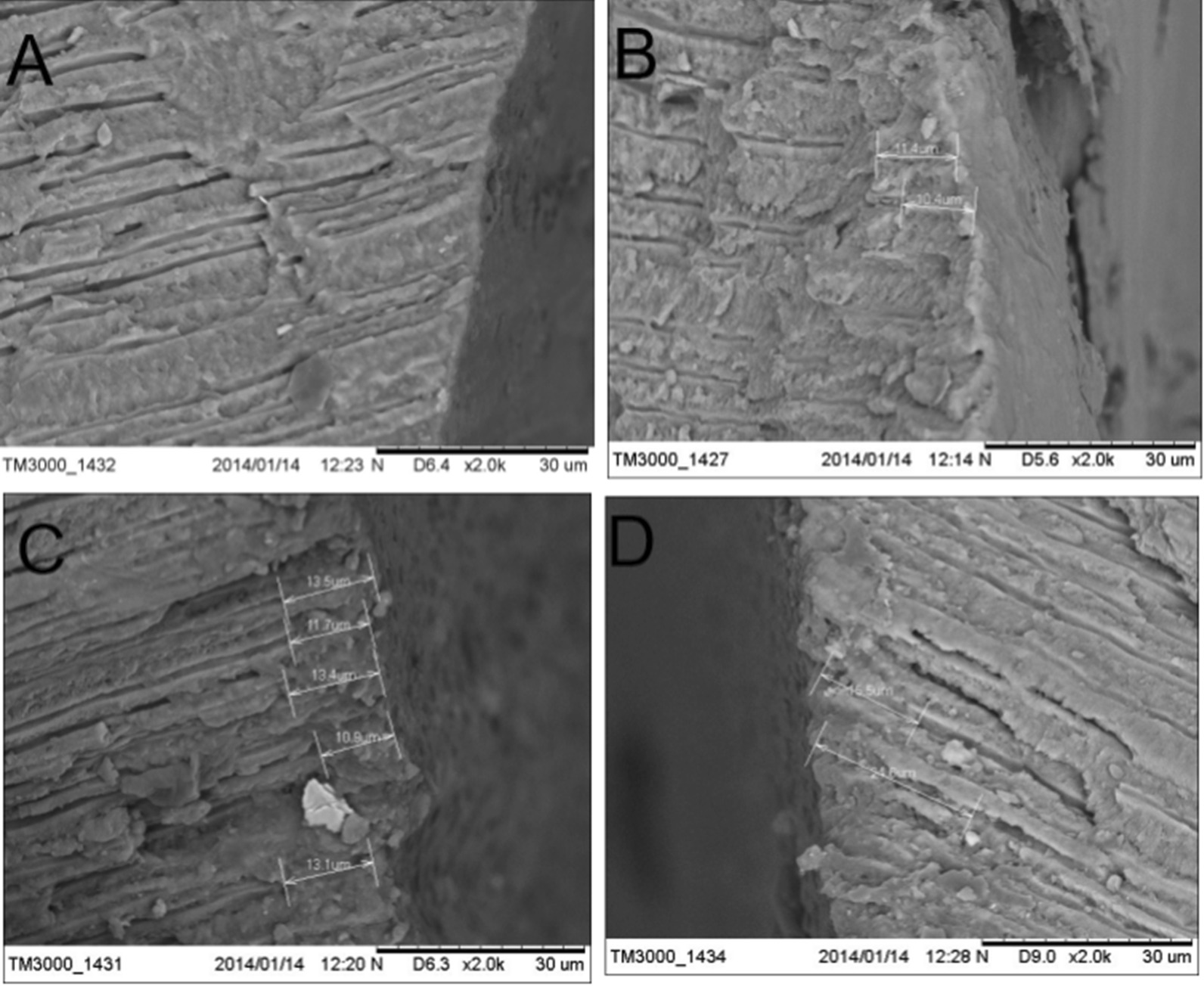
Enamel profile of the four groups after remineralization. **a** Control group; **b** Gluma group; **c** HAP group and **d** TiO_2_–HAP group

SEM imaging showed that the dentin tubules were arranged in a linear pattern and parallel to each other, and no substance was found at the opening of the dentin tubules with smooth wall in the control group (Fig. 5). The cross-sectional opening of the dentinal tubules was blocked by white matter, and the blockage was found in the dentin tubules, with a depth of about 11 µm in the Gluma group. The cross-sectional opening of the dentinal tubules was blocked by white matter, and the penetration depth of the plug into the dentin tubules was about 24 µm in the HAP group. The penetration depth of the plug into the dentin tubules was about 15 µm, and the ordered crystals grow along the dentin tubules in the TiO_2_–HAP group.

### Cytotoxicity

**Fig. 6:**
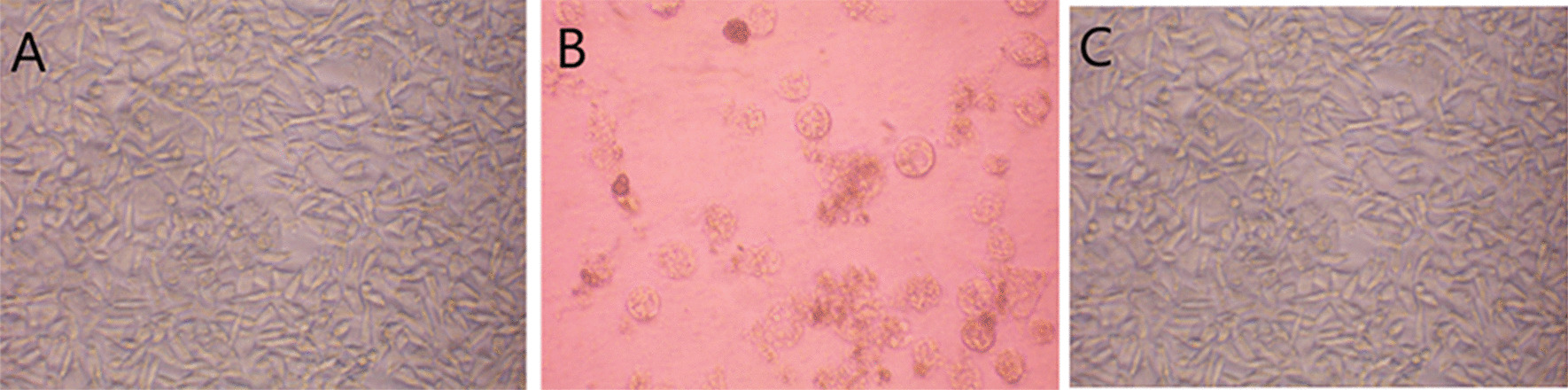
The cell imaging on the 4th day after cell culture. **a** Control group; **b** positive group; **c** 75% TiO_2_–HAP group

As shown in Table 4, the OD values of TiO_2_–HAP groups with different concentration were both higher than that of positive group (*P* < 0.05). However, there was no significant difference in OD value between control group and TiO_2_–HAP group (*P* > 0.05). Moreover, the Cytotoxicity grades of control group and TiO_2_–HAP group were both Grade 0. As shown in Fig. 6, the cell morphology was good on the 4th day after cell culture in the control and TiO_2_–HAP groups. Moreover, the density of MTT crystal in the TiO_2_–HAP group was similar to that of negative control group, showing a transparent blue purple color.

**Table 4:** Cytotoxicity of TiO_2_–HAP

Groups	OD values	RGR (%)	Cytotoxicity grade
Positive group	0.006 + 0.00	5	4
Control group	0.257 + 0.014*	100	0
75% TiO_2_–HAP group	0.286 + 0.032*	111	0
50% TiO_2_–HAP group	0.298 + 0.059*	115	0
25% TiO_2_–HAP group	0.266 + 0.041*	103	0
12.5% TiO_2_–HAP group	0.259 + 0.055*	100	0

^*^P < 0.05

## Discussion

Nanohydroxyapatite is widely incorporated in toothpaste to promote enamel remineralization as well as treat dentine hypersensitivity. In this study, we explored the effect of TiO_2_–HAP in occuluding dentin tubules by different techniques. Specific surface area and surface energy test methods were used to analyze composition and properties, and finally cytotoxicity experiments are used to verify its biological safety. Our results showed that TiO_2_–HAP can occulude the micropores on the surface of dentin in vitro without cytotoxicity.

We firstly synthesized TiO_2_–HAP by chemical precipitation method, and the phase and chemical composition of HAP carbonate were analyzed by XRD and infrared absorption spectrometer. We found that the infrared absorption at 1042, 605 and 560 cm^−1^ of the TiO_2_–HAP was consistent with the standard spectrum of HAP. The absorption peaks at 605 cm^−1^ and 560 cm^−1^ were obviously superimposed on the envelope peaks at 500–700 cm^−1^, which indicated that the sample contains both HAP and TiO_2_ [10].

The surface wettability is related to the surface energy, and the surface contact angle can objectively reflect the surface energy of the object [11]. The surface contact angle is affected by many factors. The oral cavity is often at a constant temperature. The energy reflecting the surface properties of the material rarely changes with the surface elements, so the surface energy is a better indicator for the surface wettability in coating [12]. The smaller the surface contact angle, the greater the surface energy, and the better the wettability of the object. The liquid is easy to spread on the surface and the adhesive can effectively contact the surface of the tooth. Conversely, the larger the surface contact angle, the smaller the surface energy, the poorer the wettability of the object, and the less likely it is for liquid to spread on its surface and affect the adhesion. In our study, we found that the contact angle in the TiO_2_–HAP group was smaller than that in the HAP group. Those results indicated that the wettability of TiO_2_–HAP was good.

The specific surface area of HAP is mainly affected by the total pore volume or crystal size [13]. The total pore volume is the pore volume within a predetermined pore size range, which can be determined from adsorption or desorption curves [14]. The microporous structure and large specific surface area are conducive to the attachment of biological tissues. In the present study, the specific surface area and the total pore volume in the TiO_2_–HAP group were higher than those in the HAP group. It is suggested that TiO_2_–HAP is conducive to the attachment of biological tissues. In the past few years, the main attempt is not only to control the shape of HAP powder, but also to control the crystal size, particle size distribution, porosity and crystallinity, because they have great influence on mechanical properties and biological activity [11]. In this study, mesoporous titanium dioxide HAP was compounded. The mesopores caused lattice distortion, the worse degree of crystallinity, the smaller particle size, and the increased specific surface area. Due to the unique structural state of nanoparticles, surface effects are produced, so that nanomaterials exhibit special functions such as adsorption, catalysis and biological activity [15]. Moreover, we found that the range of pore size distribution of TiO_2_–HAP was increased. It may be due to that when the size of HAP reaches the nanometer level, it will show a series of unique properties, such as high degradability and absorbability [16].

We also analyzed the surface morphology of tooth enamel. We found that the dentin tubules in the control group were clearly visible, but that of the TiO_2_–HAP group was enclosed by white matter. The results showed that material in the control group only had a certain repair effect on the surface of the glaze pillars, and there was no improvement in a large number of glaze pillar gaps. HAP without mesoporous TiO_2_ can repair the etched surface. However, HAP mainly uses the adsorption properties of nanoparticles to fill the cavities on the enamel surface, so HAP can only form a loose mineralized layer on the enamel surface. After the flower like crystals were removed by ultrasonic wave, the gap between demineralized glazes was repaired well by TiO_2_–HAP. There are a lot of mesoporous titania sheets in TiO_2_–HAP composites [17], which can cause excessive nucleation on the surface of enamel. During the process of crystal nucleation and growth, new nucleation sites are formed on the surface of enamel. These new nuclei continue to adsorb calcium and phosphorus ions in artificial saliva, grow radially and epitaxially, and finally form flower-like crystals.

Furthermore, we analyzed the profile of enamel. We found that in the TiO_2_–HAP group, the depth of occlusion was about 15 µm, and the orderly arranged crystals grew along the dentin tubules. This may be due to the fact that the negatively charged mesoporous titanium dioxide layer contained in the TiO_2_–HAP composite material can be combined with the positively charged HAP through electrostatic adsorption, which increases the orientation arrangement and adsorption binding force of TiO_2_–HAP composite particles in the demineralization zone. At the same time, the oxygen-containing functional groups on mesoporous titanium dioxide can effectively attract Ca^2+^ and PO_3_^−4^ ions from artificial saliva [18]. The orientation adsorption of HAP crystals existing in the remineralization solution and the in-situ growth of new HAP crystals interact with each other at the same time, which makes the dentin defect repaired well.

Moreover, we investigated whether TiO_2_–HAP exhibits cellular cytotoxicity in vitro after incubation of TiO_2_–HAP with cells for 4 days, we observed long spindle and polygonal and circular dividing cells similar to the control group, indicating that the cell growth was vigorous and the cell had a strong refractive index. The results showed that TiO_2_–HAP had no cytotoxicity.

## Conclusion

TiO_2_–HAP can seal the micropores on the surface of dentin in vitro. The composite has good porosity and high surface energy without obvious influence on resin binders. It has no cytotoxicity in biological safety testing. However, one potential limitation of the current study is that the durability of the coatings were not assessed, thus we could not analyze the long-term effect of TiO_2_–HAP in clinical study.

## Data Availability

The datasets used or analysed during the current study are available from the corresponding author on reasonable request.

## Abbreviations

DH: Dentine hypersensitivity
TiO_2_–HAP: Titanium dioxide composite hydroxyapatite
XRD: X-ray diffraction
SEM: Scanning electron microscope
TiO_2_: Titanium dioxide
Ca (NO_3_)_2_: Calcium nitrate
KNO_3_: Potassium nitrate
NH_3_·H_2_O: Ammonia
(NH_4_)_2_HPO_4_: Diammonium hydrogen phosphate
SD: Standard deviation
